# Targeting the IL-36 Pathway: Spesolimab as a Therapeutic for Acute Flares of Pustular Psoriasis

**DOI:** 10.7759/cureus.102134

**Published:** 2026-01-23

**Authors:** Esther Nwozo, Devon Cross, David Smith

**Affiliations:** 1 School of Medicine, Lewis Katz School of Medicine at Temple University, Philadelphia, USA; 2 Department of Dermatology, St. Luke’s University Health Network, Easton, USA

**Keywords:** biologic therapy, generalized pustular psoriasis, il-36 pathway, immunodermatology, monoclonal antibody, psoriasis, spesolimab

## Abstract

Generalized pustular psoriasis (GPP) is a rare and severe subtype of psoriasis characterized by widespread sterile pustules, erythema, and systemic inflammation. The condition is mediated by dysregulation of the IL-36 signaling pathway, which promotes excessive cytokine release and neutrophilic infiltration. Traditional therapies, including corticosteroids and immunosuppressive agents such as cyclosporine or methotrexate, often fail to achieve rapid or sustained disease control. Spesolimab, a monoclonal antibody that inhibits the IL-36 receptor, represents a novel targeted therapy approved for the treatment of acute GPP flares in adults.

We describe a 56-year-old woman with no significant past medical history who presented with diffuse erythematous and scaly plaques involving the trunk, face, groin, and extremities. Initial biopsy findings were consistent with an eczematous dermatitis, and her eruption temporarily resolved with topical corticosteroids and a prednisone taper. Five months later, she developed diffuse pustules covering 80% of her body surface area (BSA), accompanied by fever, chills, and joint pain. A repeat biopsy demonstrated subcorneal neutrophilic pustules consistent with pustular psoriasis. She was treated with cyclosporine, resulting in partial improvement to 30% body surface area involvement. The patient subsequently received a 900 mg intravenous dose of spesolimab, which produced marked improvement within two weeks, leaving less than 5% body surface area involvement. Ongoing therapy with cyclosporine and planned monthly spesolimab infusions maintained disease remission.

This case highlights the rapid efficacy and sustained control achieved with spesolimab in an acute flare of GPP refractory to conventional therapy. The results underscore the importance of IL-36 inhibition in targeting the underlying inflammatory pathway and suggest that early initiation of spesolimab may improve outcomes in patients with moderate-to-severe disease.

## Introduction

Generalized pustular psoriasis (GPP) is a rare but potentially life-threatening autoinflammatory variant of psoriasis characterized by diffuse erythema and sheets of sterile neutrophilic pustules on non-acral skin. Although it accounts for only 1%-2% of psoriasis cases, GPP carries substantial morbidity due to fever, leukocytosis, arthralgias, and metabolic disturbances, often necessitating urgent systemic therapy [1]. Its clinical heterogeneity, ranging from acute explosive flares to chronic relapsing patterns, adds to diagnostic complexity. Early lesions may resemble spongiotic or eczematous dermatitis both clinically and histopathologically, contributing to delays in recognition [2,3]. Earlier reviews have emphasized this variability and the difficulty of confidently diagnosing GPP during its early evolution [4].

Advances in molecular characterization have established IL-36 pathway hyperactivation as the central driver of GPP. Pathogenic variants in IL36RN, CARD14, AP1S3, MPO, and SERPINA3 disrupt the regulation of IL-36 receptor signaling, leading to unchecked activation of NF-κB and MAPK pathways with downstream production of IL-1β, IL-6, IL-8, and TNF-α [2]. These genetic insights increasingly support the reframing of GPP as a primarily autoinflammatory disorder rather than a variant of plaque psoriasis. Environmental and systemic inflammatory modifiers, including gut microbiome dysbiosis, may further influence flare severity and immune activation, highlighting the complex interplay between innate immunity and host factors [5]. Transcriptomic analyses demonstrate marked overexpression of IL-36-related genes in GPP lesions, reinforcing the rationale for IL-36-directed therapy [6].

Historically, treatment relied on systemic corticosteroids, cyclosporine, methotrexate, acitretin, and TNF-α/IL-17/IL-23 inhibitors, although responses were inconsistent and relapses common [4,7]. These therapeutic limitations underscore the need for an agent that could directly interrupt the upstream cytokine circuits unique to GPP. The development of spesolimab, a monoclonal antibody targeting the IL-36 receptor, represents a major therapeutic advance. In a pivotal randomized controlled trial, spesolimab induced rapid pustular clearance within 24-48 hours and sustained improvement thereafter [8]. Real-world reports have similarly documented durable clinical responses in severe or refractory flares [9].

Here, we describe a patient whose eruption initially resembled an eczematous dermatitis before evolving into fulminant pustular psoriasis. Her dramatic response to IL-36 inhibition after limited improvement with corticosteroids and cyclosporine underscores the value of repeat biopsy in evolving dermatoses and highlights spesolimab’s potential for acute GPP flares.

## Case presentation

**Figure 1:**
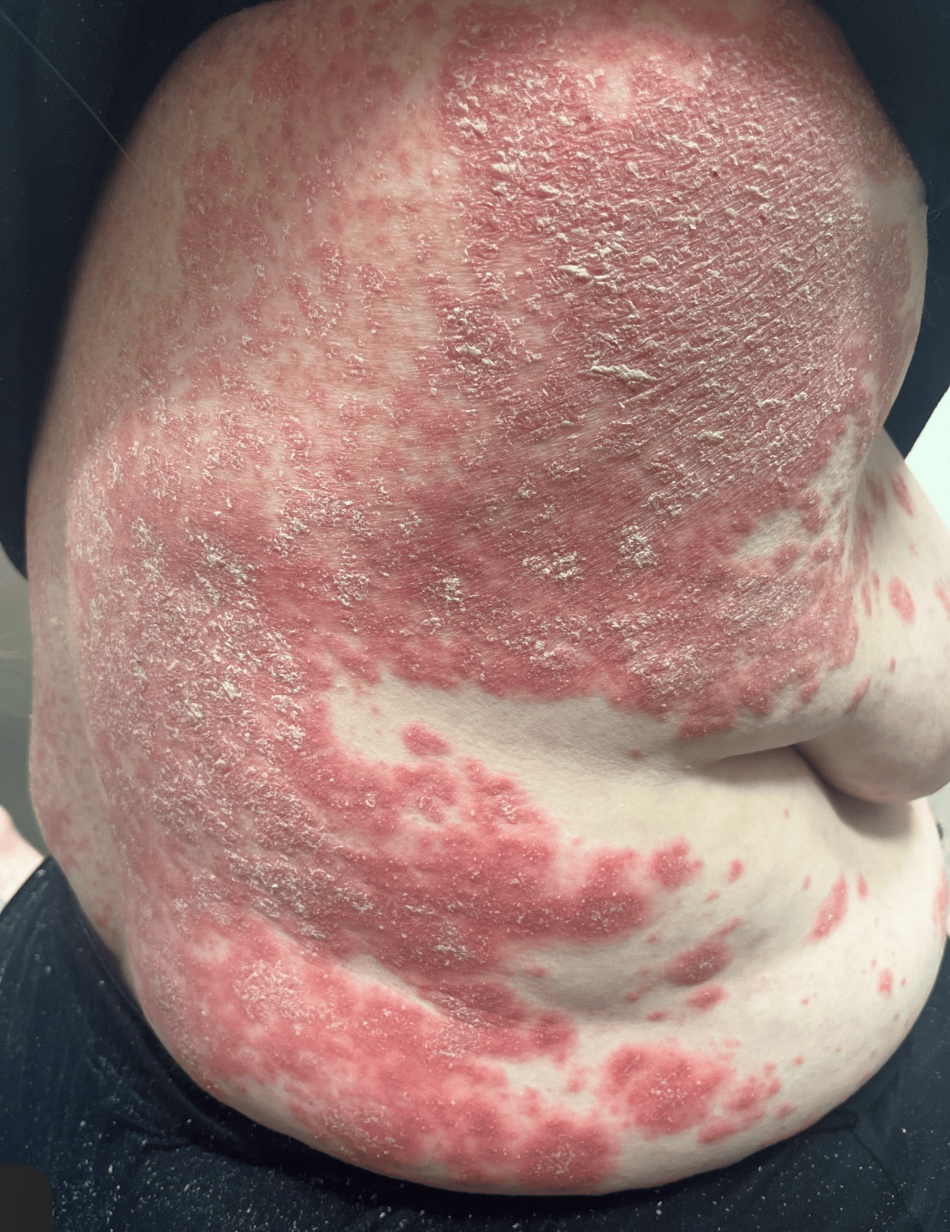
Initial presentation showing diffuse erythematous, scaly plaques on the trunk prior to pustular eruption

**Figure 2:**
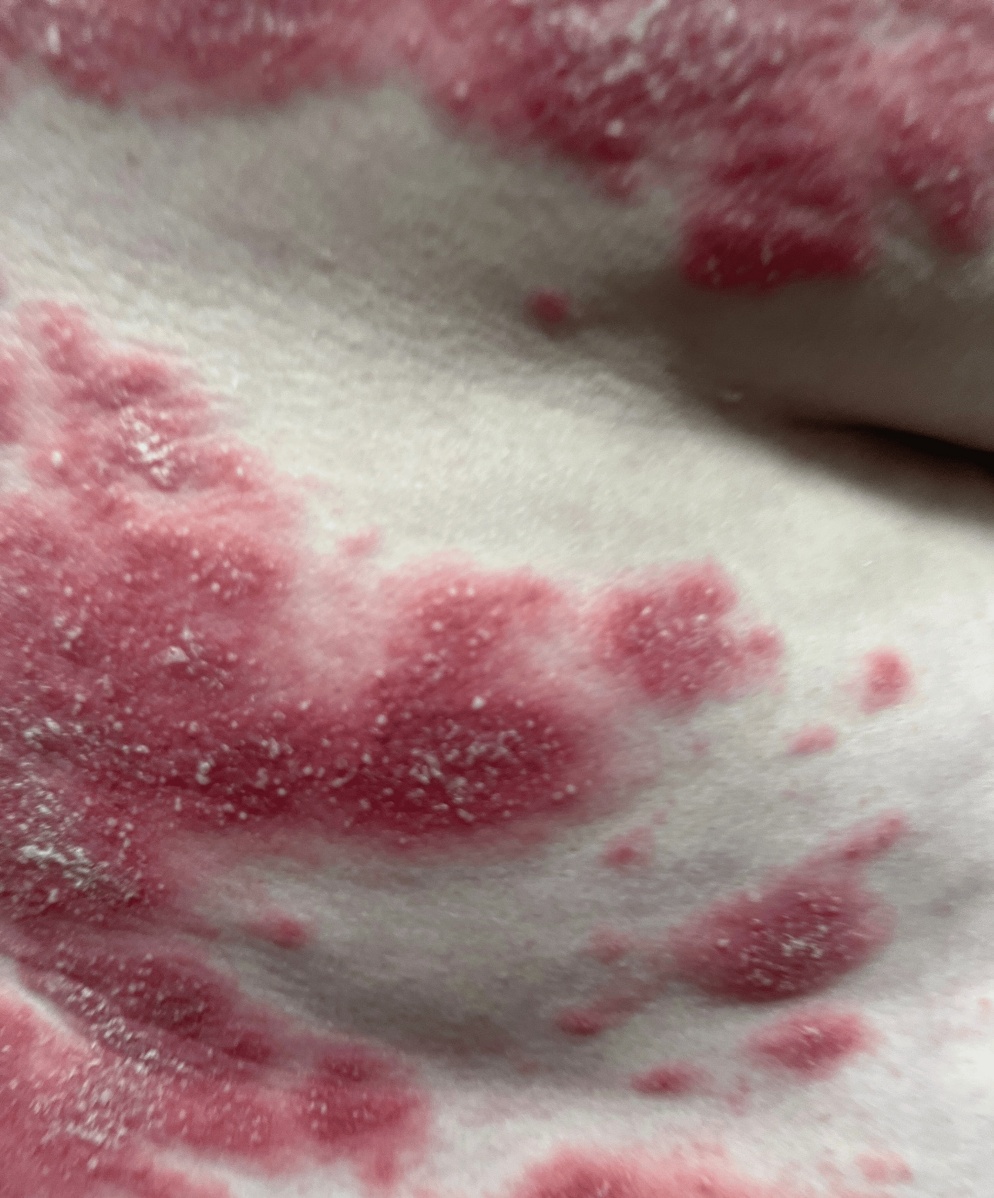
Close-up view of erythematous plaques with overlying scale on the trunk during the initial presentation

**Figure 3:**
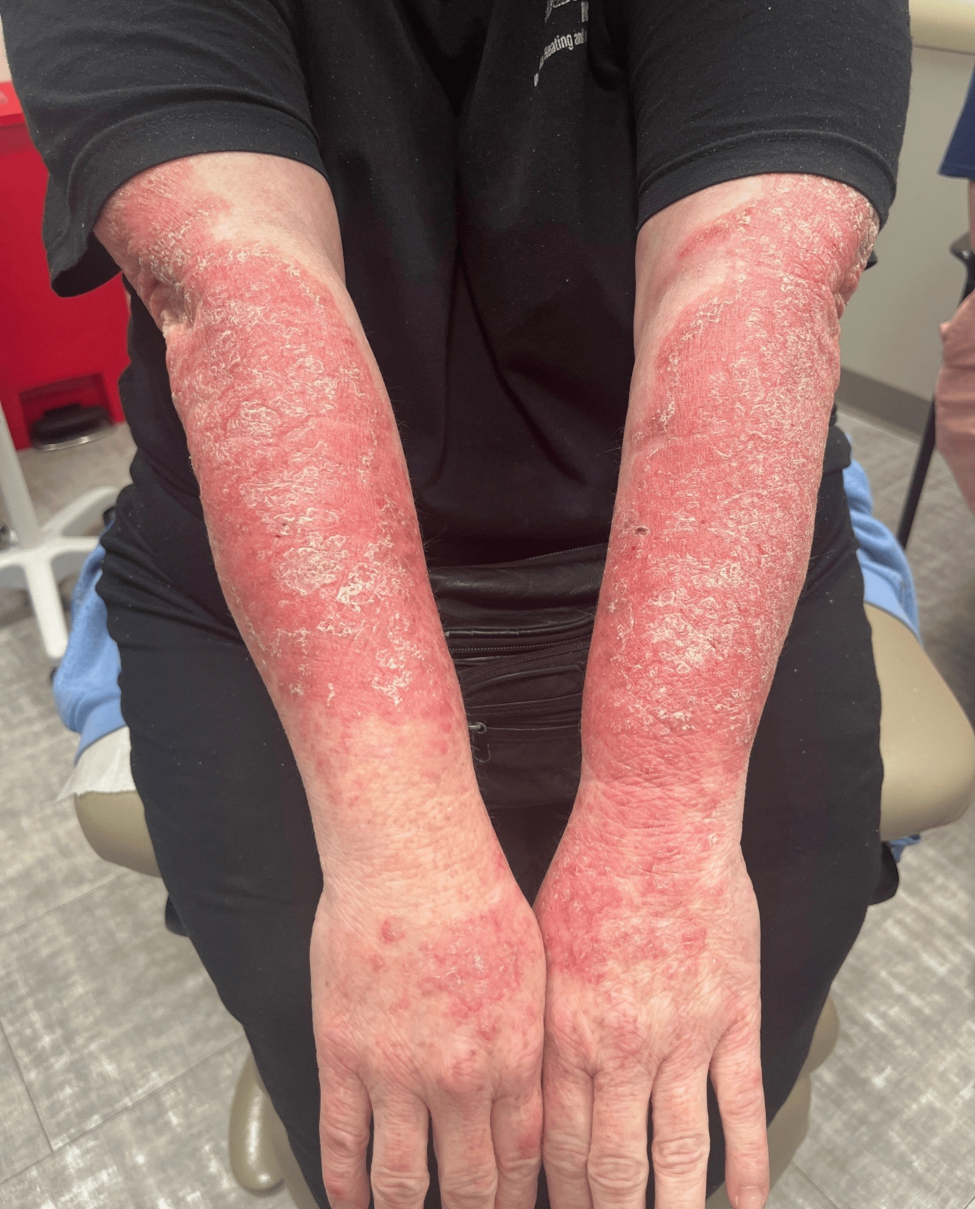
Symmetric erythematous, scaly plaques involving the bilateral upper extremities at initial presentation

We present a case of a 56-year-old woman with no significant past medical history who initially presented to the ED with pruritic, erythematous scaly plaques affecting her trunk, face, groin, and extremities (Figures 1-3). Despite treatment with Benadryl, prednisone, and clobetasol ointment, her symptoms did not improve. A punch biopsy revealed epidermal hyperplasia with mild spongiosis and multifocal serum-imbued parakeratosis, along with negative direct immunofluorescence (DIF), consistent with an eczematous process. She was subsequently prescribed triamcinolone ointment and initiated on a prednisone taper, which resolved her symptoms.

**Figure 4:**
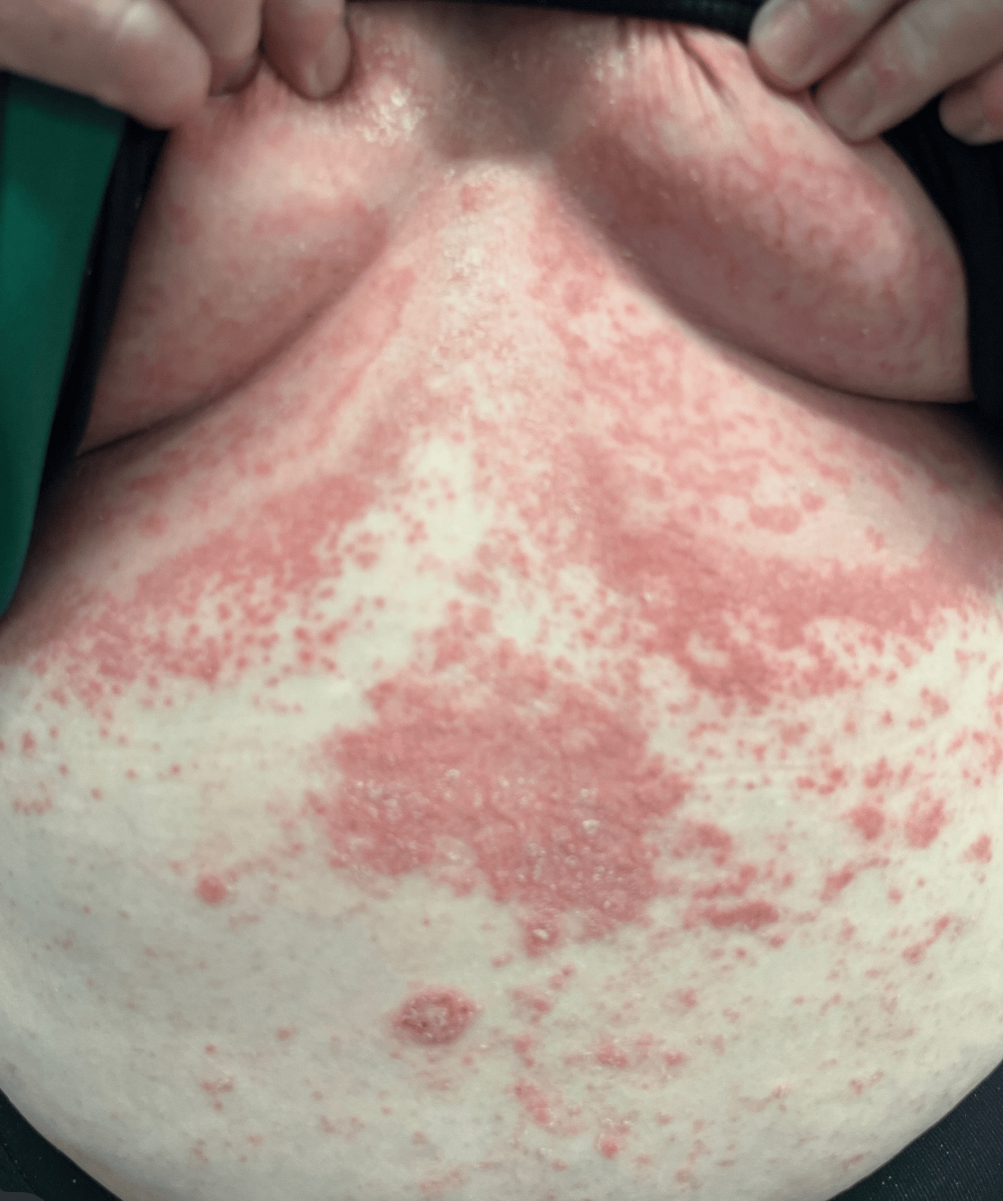
Recurrence marked by widespread erythema with scattered pustules on the trunk, accompanied by systemic symptoms

Five months later, she returned with a recurrence of the rash and new appearance of pustules accompanied by systemic symptoms including fever, chills, and joint pain (Figure 4). She had 80% body surface area (BSA) involvement. A repeat punch biopsy showed subcorneal neutrophilic pustules and negative DIF, consistent with pustular psoriasis. Cyclosporine 200 mg AM and 100 mg PM was started, along with magnesium 400 mg daily.

**Figure 5:**
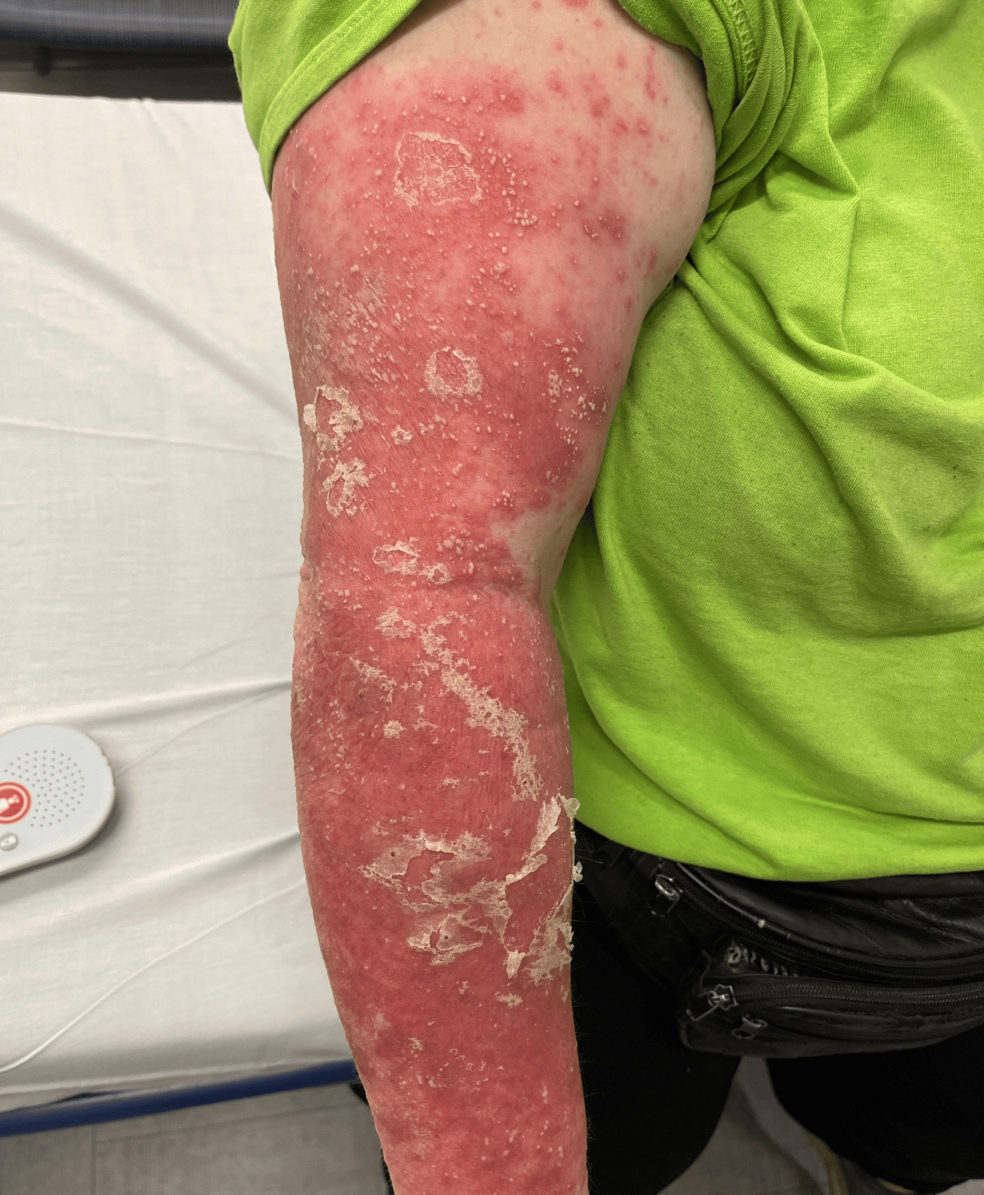
Partial clinical improvement following cyclosporine therapy, with persistent erythema, scaling, and residual pustules of the upper extremity

**Figure 6:**
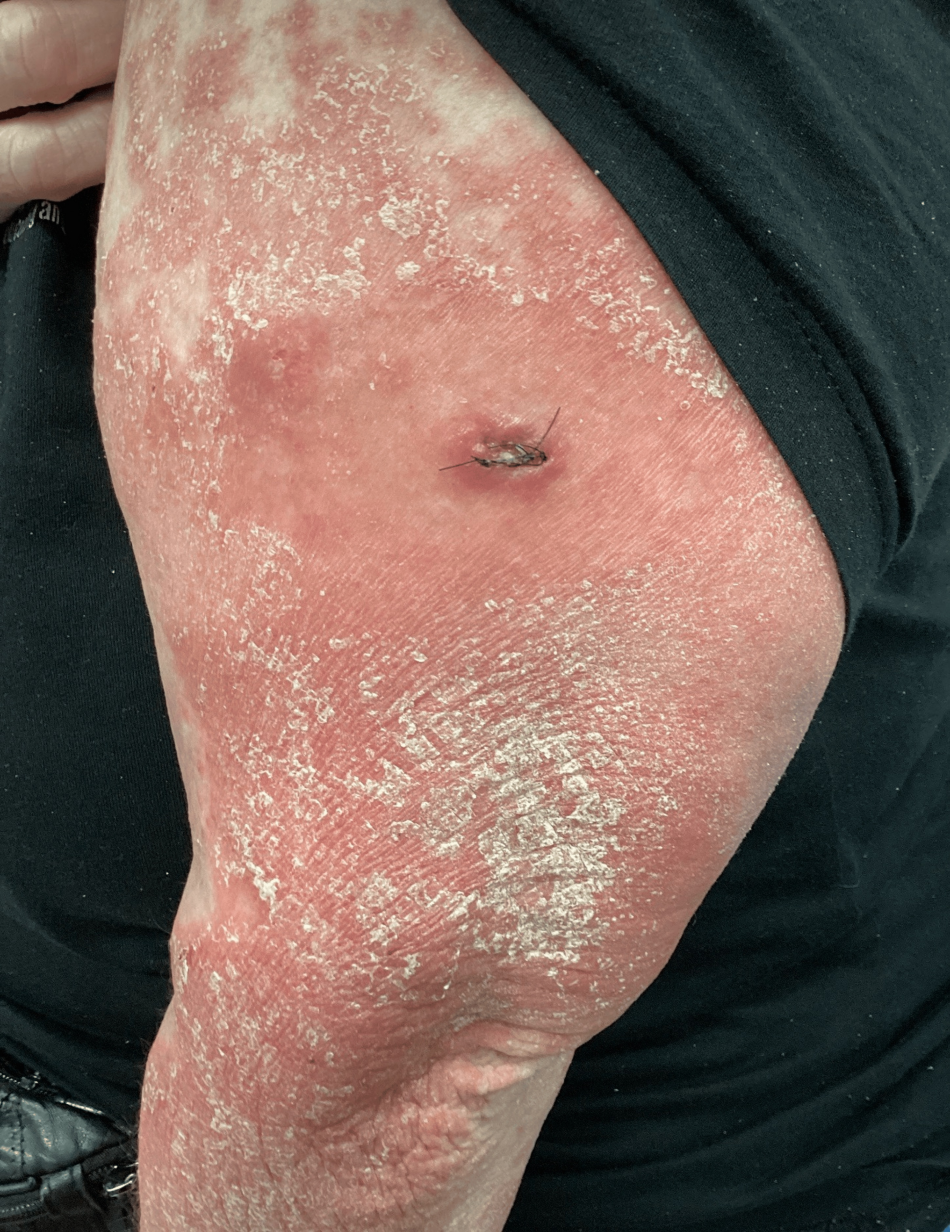
Marked reduction in erythema and scaling following IL-36 receptor inhibition with spesolimab, with residual post-inflammatory changes of the upper extremity

During follow-up one month later, she reported improvement on the cyclosporine regimen, although she still had 30% BSA involvement (Figures 5, 6). She received a 900 mg Spevigo infusion and, upon reassessment two weeks later, continued to show improvement with 5% BSA involvement. Her cyclosporine was decreased to 100 mg twice daily; however, breakthrough plaques were noted in her axilla, so the initial dose was reinstated with a plan for Spevigo injections every four weeks.

## Discussion

This case illustrates several clinically important aspects of GPP diagnosis and management, particularly the challenges posed by its variable and evolving presentation. Early in her disease course, the patient’s biopsy demonstrated spongiosis and serum-imbued parakeratosis, findings compatible with an eczematous process. This overlap between early GPP and other inflammatory dermatoses has been described previously [3,4] and reflects how early-stage or partially treated GPP may lack classic pustular features both clinically and histopathologically. Because cutaneous pathology in GPP evolves over time, reevaluating the diagnosis and pursuing repeat biopsy when the disease trajectory changes is essential for timely recognition [2]. In cases of severe or life-threatening generalized pustular psoriasis, early initiation of targeted therapy such as spesolimab has been shown to result in rapid clinical improvement and may be critical for preventing systemic complications and mortality [10].

Once pustules, fever, and extensive BSA involvement developed, cyclosporine was initiated for rapid control. Although cyclosporine remains an established option for acute GPP, its efficacy may be incomplete in IL-36-driven disease [1,4]. Persistent inflammation despite broad immunosuppression reinforces the mechanistic distinction between GPP, which is fundamentally an IL-36-mediated autoinflammatory process, and plaque psoriasis, where downstream IL-17 and IL-23 pathways predominate. This discordance underscores the need for therapies that directly interrupt IL-36-mediated signaling. Appreciating this mechanistic divide is essential because many patients with GPP are initially treated according to plaque psoriasis paradigms.

The emergence of spesolimab has significantly altered the therapeutic landscape. By blocking the IL-36 receptor, spesolimab disrupts the upstream cascade responsible for neutrophil recruitment and pustule formation. Targeting the IL-36 receptor upstream offers a conceptual advantage, interrupting the disease at its molecular ignition point rather than downstream cytokine consequences. Its biologic relevance is supported by transcriptomic data demonstrating marked IL-36 pathway activation in GPP [6], as well as genetic studies implicating IL36RN, MPO, and other variants in disease pathogenesis [2]. Clinically, spesolimab has shown rapid and profound efficacy: in the pivotal Effisayil 1 trial, more than half of patients achieved pustule clearance by day 1, with continued improvement over subsequent weeks [8]. Real-world reports further describe durable remission and meaningful improvements in quality of life among individuals with severe or refractory disease [9]. In contrast, IL-1 inhibition, while historically utilized in GPP management, acts downstream in the inflammatory cascade and may yield variable or incomplete responses, particularly in severe disease. Given that IL-36 signaling functions as a central upstream driver of neutrophilic inflammation in GPP, IL-36 receptor blockade offers a more targeted and pathophysiologically aligned therapeutic strategy compared with IL-1 inhibition, as reflected by the more rapid and consistent clinical responses observed with spesolimab [1].

Our patient’s reduction from 30% to 5% BSA involvement within two weeks of infusion parallels these findings and raises important considerations about optimal treatment strategy. Early IL-36 inhibition may limit flare progression, reduce systemic complications, and accelerate recovery [8]. Conventional agents such as cyclosporine can serve as useful bridging therapies while awaiting biologic effect or for addressing localized breakthrough inflammation, as seen in this case. Natural history studies highlight that GPP often follows a relapsing course [3], making early, mechanism-directed intervention especially valuable. Emerging evidence also suggests that some patients may require repeat dosing of spesolimab for recurrent flares, and long-term safety data and maintenance strategies continue to evolve [11]. Together, these observations emphasize the importance of flexible, individualized treatment algorithms informed by both mechanistic insight and emerging clinical evidence.

## Conclusions

In summary, this case demonstrates the diagnostic complexity of GPP, the limitations of traditional immunosuppressants in IL-36-driven disease, and the transformative impact of IL-36 receptor inhibition. Spesolimab provides rapid, targeted control of acute flares and represents a significant advancement in the management of this severe condition. As additional data emerge, a more precise framework for timing, sequencing, and maintenance strategies will develop. For now, this patient’s dramatic improvement reinforces the value of early recognition, timely reassessment, and mechanism-based therapy in optimizing outcomes for individuals with GPP.
